# Genetic Diversity and Population Structure in Seven Lipizzan Populations Based on Microsatellite Genotyping

**DOI:** 10.3390/ani16101516

**Published:** 2026-05-15

**Authors:** Biljana Rogić, Nina Moravčíková, Polonca Margeta, Ljuba Štrbac, Minja Zorc, Máté Kovács, Lubos Vostry, Radovan Kasarda, Janos Posta

**Affiliations:** 1 Faculty of Agriculture, University of Banja Luka, Bulevar Vojvode Petra Bojovica 1A, 78000 Banja Luka, Bosnia and Herzegovina; 2 Faculty of Agrobiology and Food Resources, Slovak University of Agriculture in Nitra, Tr. A. Hlinku 2, 94976 Nitra, Slovakia; nina.moravcikova@uniag.sk (N.M.); radovan.kasarda@uniag.sk (R.K.); 3 Croatian Agency for Agriculture and Food, Ulica Kardinala Alojzija Stepinca 17, 31000 Osijek, Croatia; polonca.margeta@hapih.hr; 4 Faculty of Agriculture, University of Novi Sad, Trg Dositeja Obradovića 8, 21000 Novi Sad, Serbia; ljuba.strbac@stocarstvo.edu.rs; 5 Biotechnical Faculty, University of Ljubljana, Jamnikarjeva 101, 1000 Ljubljana, Slovenia; minja.zorc@bf.uni-lj.si; 6 Doctoral School of Animal Science, University of Debrecen, 4032 Debrecen, Hungary; kovacs.mate@agr.unideb.hu; 7 Faculty of Agricultural and Food Sciences and Environmental Management, University of Debrecen, Böszörményi út 138, 4032 Debrecen, Hungary; postaj@agr.unideb.hu; 8 Faculty of Agrobiology, Food and Natural Resources, Czech University of Life Science Prague, Kamycka 129, 16500 Prague, Czech Republic; vostry@af.czu.cz

**Keywords:** genetic variability, gene flow, population subdivision, migration

## Abstract

Lipizzan horses, bred for over four centuries, represent a unique genetic, cultural and historic resource. This study assessed genetic diversity and population structure across seven European Lipizzan populations, with emphasis on the Bosnia and Herzegovina population. A total of 547 Lipizzan horses were genotyped using 12 microsatellite markers. The parameters of genetic variability, admixture level and migration rate were calculated. The mean number of alleles, effective number of alleles and Shannon’s information index were 5.78, 3.24 and 1.31, respectively. The average fixation indices were estimated to be F_IT_ = 0.05 (SE = 0.02), F_IS_ = −0.02 (SE = 0.01), and F_ST_ = 0.07 (SE = 0.01). The level of genetic diversity observed within each Lipizzan horse subpopulation shows similar fluctuations, with the lowest values observed in Bosnia and Herzegovina and the highest in the Slovak Lipizzan population. The observed heterozygosity was lower than the expected heterozygosity. The Hardy–Weinberg equilibrium was maintained in Serbia, while minor deviations occurred in other populations, most notably in Slovakia. Nei’s genetic distances were highest between Slovakia and other populations, particularly Bosnia and Herzegovina. The population structure analysis confirmed admixture involving Bosnia and Herzegovina, and the migration pattern corresponded to geographic proximity and breeding practices. These findings provide a valuable basis for developing breeding strategies to reduce inbreeding and preserve genetic diversity.

## Simple Summary

Lipizzan horses have been bred in Europe for more than 400 years and are an important part of cultural heritage, but their long history and relatively small populations raise concerns about loss of genetic diversity. This study examined the genetic variation and relationships among Lipizzan horse populations in seven countries, with special attention to the population in Bosnia and Herzegovina. DNA from 547 horses was analyzed to understand how diverse and connected these populations are. Overall, the breed shows a moderate level of genetic diversity, meaning there is still a reasonable amount of variation, although some populations are more at risk than others. The Bosnia and Herzegovina population had the lowest diversity, while the Slovak population showed the highest. The results also indicate that horses are still being exchanged between neighboring countries, which helps maintain genetic health. However, some differences between populations suggest that careful management is needed. These findings are important because they provide guidance for breeders and conservation programs to reduce inbreeding and preserve the genetic diversity of this historic breed, ensuring its long-term survival for future generations.

## 1. Introduction

The Lipizzan horse is one of the oldest European horse breeds, with a documented breeding history spanning more than 400 years. This breed possesses a unique gene pool and has significant cultural and historical value. In 2022, UNESCO recognized the uniqueness and importance of Lipizzan horses, and the tradition of Lipizzan horse breeding across eight countries was inscribed on the Representative List of the Intangible Cultural Heritage of Humanity.

The breed originated from crosses between local horses from the Slovenian Karst region and Andalusian and Neapolitan horses, as well as Arabian, Frederiksborg, and Kladruber horses. Some of these horse breeds are nearly extinct, underscoring the need for special attention to the Lipizzan horses, which represent a unique gene pool containing genetic material from several important historical breeds [1]. The development of the Lipizzan horse breed is described in detail by Dovc et al. [2].

The base population of Lipizzan horses is divided into state-owned studs. Stud books are closed, and the migration or the exchange of horses between studs is limited. Management of Lipizzan breeding is very strict and under a high level of selection. In most countries, a mating plan is approved yearly. Establishing the mating plan, it is necessary to ensure the appropriate number of stallions to maintain the optimal effective population size so that inbreeding does not occur. It is not recommended, or permitted, to mate animals with an inbreeding coefficient greater than 6.25%. Breeding goals of studs vary among countries: Austria provides horses for classical dressage, Hungary for carriage driving; Slovenia, Slovakia and Croatia for riding, while Serbia and Bosnia focus on improving the local farm horses, as well as preserving existing sire lines and mare families. Since the middle of the 20th century, private owning and breeding of Lipizzan horses has become common. Today, the global population of purebred Lipizzan horses registered within the Lipizzan International Federation (LIF) is 12,996 horses [3] with only about 15% bred at national stud farms.

In animal breeding, genetic characterization and population structure analysis are the first steps in breed conservation and may have implications for future breeding strategies and management plans [4]. Reduced genetic diversity may affect individual adaptability and fertility, thereby compromising the breed’s long-term survival probability. Microsatellite markers are very useful in examining the structure of closely related populations, and they have been widely applied in equine genetic studies [5,6,7,8,9,10], as well as in routine parentage verification [11]. Pedigree verification using microsatellites has been standard practice in Lipizzan horses for decades, especially within LIF-associated populations. Among molecular methods, microsatellites as low-cost markers have great utility in analyzing the genetic structure of interregional and large-sample horse populations, such as the Lipizzan horses. The usefulness of microsatellites for estimating genetic diversity among and within Lipizzan populations has been described in recent years [1,12]. Although high-density SNP arrays are increasingly replacing microsatellites, these markers remain valuable in molecular analysis of horse populations, especially when analyzing a large number of populations from different countries [6,13].

In the recent literature, research on genetic diversity from different Lipizzan populations, for example, Italian [14], Slovak and Slovenian [1,15], or Croatian [16], has been reported. However, the last genetic research that included Lipizzan horses from more countries was published several decades ago [12]. In Serbia, molecular genetic research on Lipizzan horses is based only on mtDNA control region analysis [17]. Furthermore, Lipizzan horses from Bosnia and Herzegovina (BH) have not previously been investigated using molecular genetic approaches. Existing studies on this population are limited to morphometric and pedigree analyses [18,19]. Given that the Vučijak stud farm from BH was a full member of LIF until 2022, assessing the genetic position of the BH population relative to other European Lipizzan populations is of particular interest.

The main aim of this study was to assess the current level of genetic diversity within and among Lipizzan horse populations from seven countries, including BH. Additionally, the aim was to analyze and geographically illustrate molecular genetic relationships between subpopulations, with particular emphasis on BH and finally, to provide insights into population structure, differentiation, and gene flow among Lipizzan studs.

## 2. Materials and Methods

### 2.1. Population Data Collection

In the present study, DNA-based parentage test results from 547 Lipizzan horses originating from seven different countries were analyzed. The distribution of samples was as follows: 159 horses from Bosnia and Herzegovina (BH), 119 from Serbia (SRB), 51 from Croatia (CRO), 46 from the Czech Republic (CZ), 61 from Slovakia (SK), 50 from Hungary (HUN) and 61 from Slovenia (SLO). DNA-based parentage testing data were obtained from collaborating National Studs and National Horse Breeders Associations. Only final microsatellite genotypes were available for the analysis, and no direct laboratory work was performed within the scope of this study. Genotyping had been previously carried out in accredited laboratories as part of routine parentage control, using a panel of 12 microsatellite markers (AHT4, AHT5, ASB2, HMS2, HMS3, HMS6, HMS7, HTG10, HTG4, HTG6, HTG7 and VLH20), recommended by the International Society for Animal Genetics (ISAG) and the Equine Genetics Standing Committee. Although detailed PCR conditions and genotyping procedures were not available, all laboratories followed standardized protocols for equine parentage testing. Quality control and allele scoring were performed according to routine laboratory procedures.

### 2.2. Genetic Diversity

The mean number of alleles (Na), number of effective alleles (Ne), observed (Ho) and expected heterozygosity (He), Shannon’s information index (I), as well as the number of private alleles (NPA) were used to estimate genetic variability within and between populations. The analysis of molecular variance was conducted using GenAlEx 6.5 [20]. Deviations from the Hardy–Weinberg equilibrium were tested using locus-by-locus Chi-square test with Bonferroni correction. Wright’s fixation indices (F_IS_, F_IT_ and F_ST_) were evaluated. GenAlEx 6.5 [20] was used to calculate these parameters.

### 2.3. Population Structure and Genetic Relationship

Genetic differentiation between individuals and between populations was evaluated using Nei’s genetic distance and on population level based on F_ST_ coefficient. Principal coordinate analysis (PCoA), based on the matrix of individual genetic distance, was performed to reveal major patterns of genetic variability and clustering among Lipizzan populations. The calculation was done using GenAlEx 6.5 [20]. The population structure was inferred using a Bayesian clustering approach proposed by Pritchard et al. [21] implemented in the STRUCTURE 2.3.4 software. The analysis was performed using an admixture model with correlated allele frequencies, with a burn-in period of 100,000 followed by 1,000,000 Markov chain Monte Carlo iterations. For each tested number of clusters (K = 1–10), 10 independent runs were performed. The optimal K value was determined based on evaluation of the log probability of delta K (ΔK), according to Evano et al. [22] using STRUCTURE SELECTOR [23]. The visualization of admixture patterns among Lipizzan populations was done using CLUMPAK 1.1. [24]. Recent migration among populations was evaluated using the Bayesian MCMC method implemented in the BAYESASS program v. 1.3 [25]. The migration rate was visualized using the circlize package in R [26].

## 3. Results

### 3.1. Genetic Diversity

Genetic diversity parameters used to evaluate genetic diversity across all seven Lipizzan populations are presented in Table 1. All 12 microsatellite loci analyzed were polymorphic, and their alleles were present or shared across all seven Lipizzan populations. The average number of alleles per locus was 5.78, ranging from 4.57 (HMS7) to 6.86 (HMS). The lowest number of effective alleles was observed at the HTG4 locus (2.40), while the highest was recorded at the ASB2 locus (3.90), with an overall mean Ne of 3.24. Shannon’s information index ranged from 1.03 (HMS6) to 1.57 (HMS7). The observed heterozygosity ranged from 0.58 (HTG4) to 0.77 (HMS7 and AHT4), with an average of 0.68. The highest gene diversity was observed at the HMS7 locus (0.75), and the lowest at HTG4 (0.58), with a population average of 0.67.

The average inbreeding coefficient between individuals within populations, as expressed by the F_IS_ index, was −0.02. The inbreeding coefficient between individuals in population (F_IT_) varied from −0.01 (HTG7) to 0.18 (HTG4), with an average value of 0.05. The mean F_ST_ index was 0.07, suggesting that most genetic variation occurs within rather than among Lipizzan populations. The HTG10 and HTG4 loci exhibited higher F_IT_ and F_ST_ values compared to other markers.

Genetic diversity parameters for individual Lipizzan subpopulations are presented in Table 2. The mean number of alleles ranged from 5.00 (BH) to 7.25 (SK). Similar variation patterns were observed for effective allele number (Ne), Shannon’s information index (I), and both observed (Ho) and expected (He) heterozygosity. Overall, lower diversity values were recorded in the BH population, while the SK population exhibited the highest diversity. No private alleles were detected in the SRB Lipizzan populations. For BH, CZ, and HUN, the number of private alleles was 0.08, followed by SLO (0.17), CRO (0.25), with the highest value observed in SK population (1.42). The number of different alleles with a frequency of ≥5% ranged from 3.67 (BH) to 4.17 (CZ), while the No. LComm Alleles (≤50%) ranged from 0.75 (BH, HUN) to 1.17 (SRB). In all seven Lipizzan populations, Ho values were slightly lower than He values, indicating no evidence of heterozygote deficiency.

Hardy–Weinberg equilibrium (HWE) was assessed across the 12 loci for all seven Lipizzan populations (Table 3). No significant deviations from HWE were observed in the SRB population. In the BH, HUN, and SLO populations, one locus showed significant deviation (*p* < 0.05), while in the CRO population, one locus showed highly significant deviation (*p* < 0.001). Four microsatellite loci deviated from HWE in the CZ population, and the highest number of deviations was observed in the SK population (five loci).

### 3.2. Population Structure and Genetic Relationship

Analysis of molecular variance (AMOVA) revealed that the subdivision of Lipizzan horse populations can explain 7% of the total genetic variation, while 93% was distributed within individuals across all populations. No significant variation was detected among individuals within populations (0%). Genetic differentiation among and within populations was further tested using Nei’s distances and F_ST_ coefficient (Table 4). The highest genetic distances (Nei’s) were observed in the Slovak (SK) population and the other populations, ranging from 0.331 between SK and CZ to 0.485 between SK and BH. The lowest genetic distance was recorded between the CRO and CZ populations (0.061). F_ST_ values show similar results, with the lowest differentiation observed between CRO and CZ (0.013), and the highest between SK and BH Lipizzan population (0.084).

**Figure 1:**
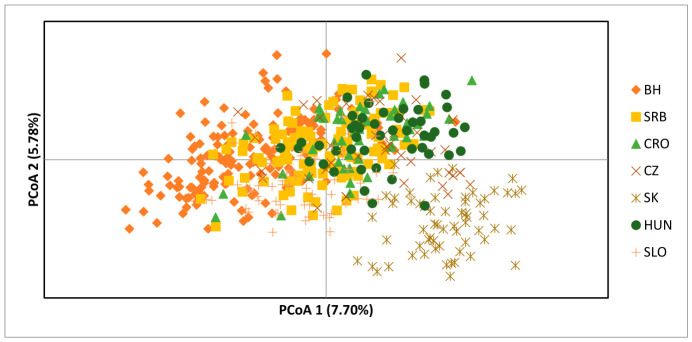
Genetic clusters determined using discriminant analysis of principal coordinate analysis (PCoA).

Clustering results based on principal coordinate analysis (PCoA) are presented in Figure 1. When comparing the entire Lipizzan population, some differences between populations were observed, but overall genetic admixture was evident.

The discriminant analysis of principal components indicated the division among individuals into the cluster in relation to country of origin. The BH populations had the main overlap with CRO and SRB, as well as SLO, while there is less overlap with SK, CZ and HUN Lipizzan populations.

**Figure 2:**
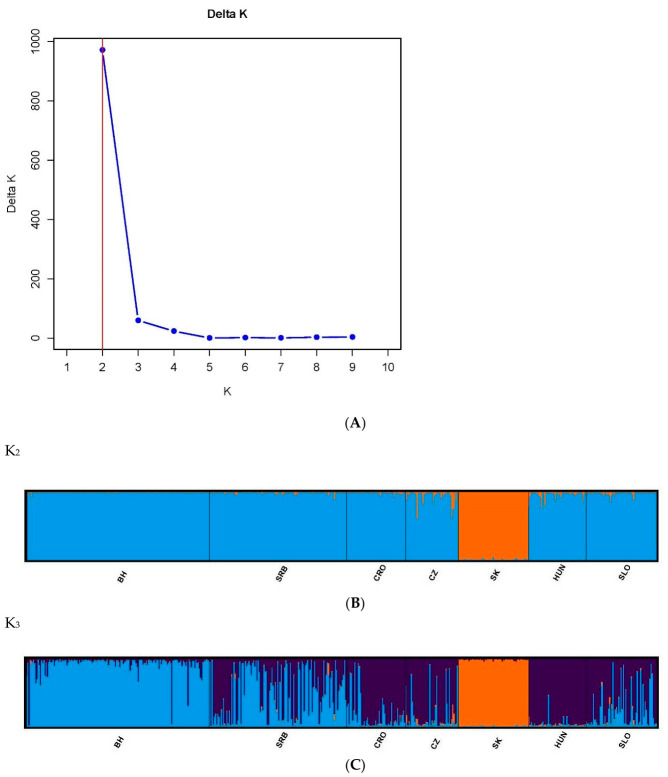
The highest likelihood and Delta K were observed for K = 2 (**A**). Hierarchical plot representing results from Bayesian assignment analysis implemented in STRUCTURE, with the number of genetic clusters K = 2 (**B**) and K = 3 (**C**). Population group labels: BH—Bosnia and Herzegovina, SRB—Serbia, CRO—Croatia, CZ—Czesh Republic, SK—Slovakia, HUN—Hungary and SLO—Slovenia.

The population structure of the seven Lipizzan populations was further analyzed using a Bayesian clustering approach implemented in STRUCTURE, assuming correlated allele frequencies. The results of Delta K indicated that the optimal number of genetic clusters representing the most similar ancestral breeds was K = 2 (Figure 2A). The mixed colors and proportional lengths represent the admixture level for the subpopulations of K between 2, 3 and 6. First level of clustering (K = 2) separated Slovakian population from the other Lipizzan populations (Figure 2B). At the additional level of K  =  3, the identified subpopulations were subsequently sub-structured (Figure 2C).

**Figure 3:**
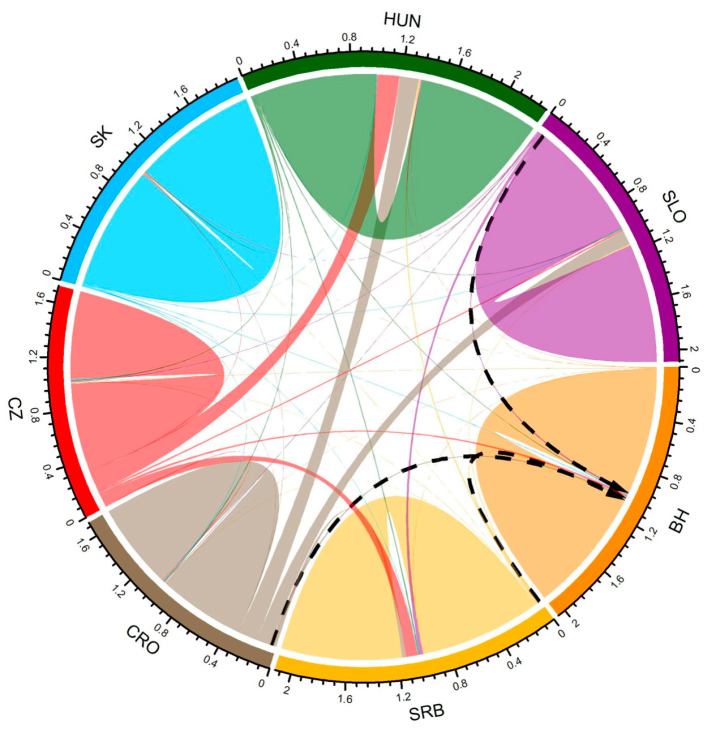
Migration rate detected using BayesAss between seven Lipizzan populations.

Migration rates between seven Lipizzan populations were estimated using the BayesAss model (Figure 3, Table S1). In terms of the BH population, the highest migration rates were observed with SRB Lipizzan populations (0.005), while the lowest involved the SK population (0.002). The CRO and CZ Lipizzan populations have the highest migration rates with HUN (0.1516 and 0.1732, respectively), and the lowest with SK (0.0059 and 0.0067). The highest migration rate for the SK population was recorded with CZ population (0.0067), and the lowest with SRB population (0.0049). The HUN population showed the highest migration with CZ (0.0109) and the lowest with SK population (0.0058). Finally, the SLO population has the highest migration with SRB (0.0268) and the lowest with the SK population (0.0050).

## 4. Discussion

The last studies that utilized microsatellites that included larger populations of Lipizzaner horses date back several decades [12,27]. This paper represents the first comprehensive study of the genetic diversity and population structure of the Lipizzan population across seven countries after several decades.

According to FAO recommendations, microsatellite markers with more than four alleles are suitable for assessing genetic diversity [28]. In this study, all 12 loci exhibited higher Na values recommended by FAO, confirming the suitability of the selected markers for analyzing genetic diversity in the Lipizzan population. The results for the mean number of alleles, Shannon’s index, and observed and expected heterozygosity are consistent with findings from previous studies on Lipizzan horses [1,12,15]. Compared with other horse breeds, the levels of genetic diversity observed in this study are slightly lower than those reported for Arabian, Hucul, Nonius, and Danubian horse breeds [9,13,29,30] but are comparable to those found in other native horse breeds [3].

The lower genetic diversity observed in the Lipizzan breed compared to other horse breeds can likely be attributed to the limited number of stallions and mares used in breeding, as well as the restricted exchange of breeding animals between stud farms in different countries. Genetic diversity within individual subpopulation showed low levels of variation, consistent with their shared historical background. Partially low genetic diversity was recorded for the BH population. The stud farm Vučiak has gone through a difficult period in recent decades. In particular, the period during and after the Civil War in the 1990s put the stud farm Vučiak in a difficult position. At that time, they faced a lack of funds for the horses’ subsistence needs, as well as a blockade of cross-border cooperation with other stud farms. The consequence was that for decades, Lipizzan breeding at the stud farm Vučijak was carried out exclusively within the population. The last two stallions were imported in 2006 from the stud farm Lipica (Slovenia), and there have been no new stallion imports until now. From a genetic point of view, this indicated that the BH Lipizzan population was exposed to bottlenecks and genetic drift, as well as inbreeding, which could ultimately lead to a decrease in genetic diversity.

The higher number of private alleles detected in the Slovak Lipizzan population indicates the presence of population-specific genetic variation not observed in the other studied populations. This pattern may reflect a combination of historical factors, including partial reproductive isolation, genetic drift, and specific population management practices, as well as the preservation of particular founder lineages. Given the structured breeding history of the Lipizzan breed, the Slovak population may retain unique genetic components linked to particular sire lines that have been reduced or are absent elsewhere. At the same time, it should be noted that the number of private alleles can be influenced by sample size and marker set and therefore should be interpreted with caution.

Deviation from HWE was observed for one (BH, HUN, SLO and CRO), four (CZ) and five (SK) loci. This deviation can be interpreted as a result of small populations (genetic drift), mating with relatives (inbreeding), as well as the intensive artificial selection, which is a characteristic of Lipizzan breeding.

Measures of genetic differentiation, including F_ST_ and Nei’s genetic distance, indicated low genetic differentiation between subpopulations, as expected given their common origin and similar breeding programs of the analyzed populations. The F_ST_ values were below 0.05 for all Lipizzan populations, except for the SK population, which were slightly higher, ranging from 0.055 to 0.084. These F_ST_ values indicated 1.3 to 8.4% of the microsatellite variability, which is explained by population subdivision, while the majority of variation is explained by variation within subpopulations. A similar range (2.1–8.0%) was reported by Achmann et al. [12], indicating only minor changes in genetic differentiation over the past two decades. The obtained results indicated that inbreeding depression is not a significant concern in the Lipizzan horse population, as the F_IT_ values for all microsatellite loci were close to zero (0.05). The indicator of inbreeding between individuals (F_IS_) in a population ranged from −0.09 to 0.05, and was negative for most loci (except HMS2, HMS3 and VLH20). This indicates low levels of inbreeding and no substantial heterozygote deficiency across the seven Lipizzan subpopulations. Negative F_IS_ values indicate an excess of heterozygotes due to selective breeding that avoids matings between closely related individuals. Similar results were described by Achmann et al. [12], indicating that, over the past two decades, the differentiation between Lipizzan subpopulations has changed slightly. Grilz-Seger et al. [31], reported that genomic inbreeding, described by fROH, in the Lipizzan breed (fROH = 0.13) was also lower than expected from long-term stud farm breeding at small census, indicating that it was lower comparing with Shagya and Purebred Arabian horses.

Population structure results revealed some differentiation of the Slovak Lipizzan population from the others. Admixture level between BH and other Lipizzan populations was confirmed. The main overlap was detected with SRB, CRO and SLO populations, which is in accordance with the studbook and imports of stallions and mares at the stud farm Vučijak. Historically, Lipizzan horses originated in Lipica during the Austro-Hungarian Empire. As breeding expanded, other stud farms were formed after the First and Second World Wars. The exchange of stallions and mares between stud farms is always present, which explains the different migration rates. Migration rates estimated using the BayesAss model showed different migration patterns among populations. The obtained results are consistent with the geographical distribution of populations and their breeding goals. Higher migration rates were observed between populations from neighboring countries, whereas more distant populations showed reduced gene flow. For example, regarding the BH population, the highest migration rates were observed with SLO, SRB and CRO. These were expected because these countries were recently part of the same state, the Former Republic of Yugoslavia and confirm the historical gene flow. Similarly, the highest migration rate between SK and CZ populations, as members of the former Czechoslovakia, also confirms historical gene flow. The CRO population has the highest migration rate with HUN. In recent decades, Croatia (especially the stud farm Đakovo) has imported more stallions from Hungary due to shared breeding goals and geographic proximity, which explains the obtained result. The estimated migration rates were also consistent with the observed levels of genetic differentiation: populations with greater differentiation exhibited lower migration rates, and vice versa.

The small number of microsatellite loci used and the limited genome coverage cannot detect the fine-scale genetic structure. The next step should be to use SNP or resequencing technology in future studies of the genetic diversity and structure of the Lipizzan horse population.

## 5. Conclusions

In conclusion, the results of this study provide new insights into the genetic diversity and population structure of seven Lipizzan horse populations. The findings confirm low levels of differentiation among subpopulations, consistent with the shared history of the breed and ongoing exchange of horses (export/import) between countries. Genetic diversity within subpopulations showed limited variation, indicating stable diversity parameters comparable to those reported approximately two decades ago. Furthermore, the results indicate low levels of inbreeding and no substantial heterozygous deficiency across the seven Lipizzan subpopulations. Overall, these findings demonstrate that genetic variability in the Lipizzan breed has been well preserved, confirming the proper management of breeding programs and mating plans implemented in the stud farms. However, some differences between populations suggest that it is necessary to continue with proper management, with the aim to preserve the genetic diversity of the Lipizzan horses for future generations. Future studies employing genome-wide sequencing data are recommended to provide a more detailed understanding of the genetic diversity and population structure in this breed.

## Figures and Tables

**Table 1:** Characteristics of the 12 microsatellite loci analyzed in seven Lipizzan populations.

	Na	Ne	I	Ho	He	F_IT_	F_IS_	F_ST_
AHT4	5.28	3.78	1.42	0.77	0.73	−0.03	−0.06	0.02
AHT5	4.86	3.46	1.33	0.71	0.71	0.04	−0.01	0.05
ASB2	6.29	3.9	1.51	0.75	0.74	0.02	−0.02	0.04
HMS2	6.86	3.02	1.35	0.66	0.67	0.03	0	0.03
HMS3	6	3.89	1.51	0.7	0.74	0.09	0.05	0.04
HMS6	4.57	2.53	1.03	0.64	0.6	−0.05	−0.07	0.02
HMS7	6.86	4.13	1.57	0.77	0.75	0.02	−0.03	0.04
HTG10	6.71	3.05	1.33	0.66	0.66	0.13	−0.01	0.13
HTG4	5.71	2.4	1.08	0.58	0.58	0.18	−0.01	0.19
HTG6	5.43	2.5	1.11	0.6	0.59	0.05	−0.01	0.07
HTG7	4.71	3.01	1.17	0.71	0.65	−0.01	−0.09	0.08
VLH20	6.14	3.27	1.36	0.68	0.68	0.09	0.01	0.08
Mean (SE)	5.78 (0.45)	3.24 (0.19)	1.31 (0.06)	0.68 (0.03)	0.67 (0.02)	0.05 (0.01)	−0.02 (0.02)	0.07 (0.01)

Notes: Number of identified alleles per locus (Na), number of effective alleles (Ne), Shannon’s information index (I), observed (Ho) and expected (He) Heterozygosity, F_IT_ (inbreeding coefficient), F_IS_ (inbreeding coefficient of an individual relative), F_ST_ (fixation index).

**Table 2:** The main parameters of the genetic diversity of the seven Lipizzan populations (average values and SE).

	BH	SRB	CRO	CZ	SK	HUN	SLO
Na	5.00 (0.25)	6.08 (0.48)	5.50 (0.45)	5.75 (0.30)	7.25 (0.51)	5.58 (0.23)	5.33 (0.31)
Ne	2.95 (0.25)	3.28 (0.24)	3.24 (0.21)	3.31 (0.21)	3.70 (0.26)	3.19 (0.17)	3.05 (0.17)
I	1.19 (0.08)	1.32 (0.07)	1.31 (0.08)	1.35 (0.06)	1.47 (0.06)	1.30 (0.06)	1.27 (0.05)
Ho	0.63 (0.03)	0.68 (0.02)	0.68 (0.02)	0.69 (0.02)	0.72 (0.02)	0.68 (0.02)	0.67 (0.02)
He	0.63 (0.03)	0.68 (0.02)	0.67 (0.02)	0.68 (0.02)	0.71 (0.02)	0.68 (0.02)	0.66 (0.02)
NPA	0.08 (0.08)	0.00 (0.00)	0.25 (0.18)	0.08 (0.08)	1.42 (0.60)	0.08 (0.08)	0.17 (0.11)
Na Freq. ≥ 5%	3.67 (0.31)	3.75 (0.30)	3.75 (0.33)	4.17 (0.32)	4.00 (0.33)	4.08 (0.34)	3.83 (0.24)
No. LComm Alleles (≤50%)	0.75 (0.22)	1.17 (0.32)	0.92 (0.29)	1.08 (0.34)	0.92 (0.29)	0.75 (0.35)	1.00 (0.33)

Notes: Mean number of alleles (Na), number of effective alleles (Ne), Shannon’s information index (I), observed (Ho) and expected (He) heterozygosity, number of private alleles unique to a single population (NPA) and Na freq. ≥ 5% (mean number of alleles for which the frequency is equal to or not less than 5%), No. LComm Alleles (≤50%) = No. of Locally Common Alleles (Freq. ≥ 5%) Found in 50% or Fewer Populations in seven Lipizzan populations.

**Table 3:** Hardy–Weinberg (HWS) equilibrium test for all studied microsatellite loci in the seven Lipizzan populations.

Locus	BH	SRB	CRO	CZ	SK	HUN	SLO
AHT4	0.495	0.796	0.400	0.007 **	0.393	0.400	0.350
AHT5	0.670	0.207	0.734	0.341	0.830	0.212	0.044 *
ASB2	0.860	0.304	0.000 ***	0.495	0.022 *	0.111	0.718
HMS2	0.047 *	0.678	0.345	0.004 **	0.007 **	0.852	0.962
HMS3	0.734	0.217	0.721	0.075	0.000 ***	0.019 *	0.745
HMS6	0.914	0.789	0.072	0.443	0.000 ***	0.996	0.308
HMS7	0.081	0.754	0.570	0.005 **	0.905	0.670	0.938
HTG10	0.789	0.746	0.414	0.552	0.007 **	0.613	0.906
HTG4	0.894	0.718	0.880	0.604	0.000 ***	0.728	0.998
HTG6	0.900	0.953	0.912	0.963	0.981	0.999	0.183
HTG7	0.171	0.335	0.328	0.204	0.083	0.491	0.074
VLH20	0.487	0.232	0.803	0.001 ***	0.469	0.116	0.059

Significant *p*-values (*p* < 0.05 *, *p* < 0.01 **, *p* < 0.001 ***).

**Table 4:** Pairwise population matrix of Nei’s genetic distances (below the diagonal) and pairwise population F_ST_ values (above the diagonal) between seven Lipizzan populations.

	BH	SRB	CRO	CZ	SK	HUN	SLO
BH		0.024	0.031	0.035	0.084	0.049	0.026
SRB	0.092		0.020	0.016	0.066	0.028	0.024
CRO	0.128	0.092		0.013	0.069	0.020	0.028
CZ	0.142	0.073	0.061		0.055	0.016	0.029
SK	0.485	0.404	0.429	0.331		0.067	0.072
HUN	0.217	0.135	0.091	0.071	0.412		0.042
SLO	0.104	0.103	0.124	0.132	0.425	0.196	

## Data Availability

The datasets used and analyzed during the current study are available from the corresponding authors upon reasonable request.

## Supplementary Materials

The following supporting information can be downloaded at: https://www.mdpi.com/article/10.3390/ani16101516/s1. Table S1: Migration rate detected using BayesAss between seven Lipizzan populations.
