# Supplementary material for: Genetic Diversity and Population Structure in Seven Lipizzan Populations Based on Microsatellite Genotyping
Source: Animals (Basel). 2026 May 15;16(10):1516. doi: 10.3390/ani16101516 (PMC13203804; doi:10.3390/ani16101516)
Supplement: Supplementary file 1 [file animals-16-01516-s001.zip › animals-4258348-supplementary.pdf]

Table S1. Migration rate detected using BayesAss between seven Lipizzan populations

	**BH**	**SRB**	**CRO**	**CZ**	**SK**	**HUN**	**SLO**
**BH**	0.9796	0.0050	0.0022	0.0040	0.0020	0.0034	0.0038
**SRB**	0.0190	0.9495	0.0044	0.0039	0.0026	0.0135	0.0071
**CRO**	0.0170	0.0289	0.6764	0.0064	0.0059	0.1516	0.1138
**CZ**	0.0181	0.0929	0.0082	0.6870	0.0067	0.1732	0.0140
**SK**	0.0051	0.0049	0.0061	0.0067	0.9673	0.0050	0.0049
**HUN**	0.0076	0.0103	0.0087	0.0109	0.0058	0.9484	0.0082
**SLO**	0.0144	0.0268	0.0052	0.0059	0.0050	0.0071	0.9356
